# Redox Property Tuning in Bipyridinium Salts

**DOI:** 10.3389/fchem.2020.631477

**Published:** 2021-01-27

**Authors:** Zuzana Burešová, Milan Klikar, Petr Mazúr, Michaela Mikešová, Jaroslav Kvíčala, Tomas Bystron, Filip Bureš

**Affiliations:** ^1^ Institute of Organic Chemistry and Technology, Faculty of Chemical Technology, University of Pardubice, Pardubice, Czechia; ^2^ Department of Chemical Engineering, Faculty of Chemical Engineering, University of Chemistry and Technology Prague, Prague, Czechia; ^3^ Department of Organic Chemistry, Faculty of Chemical Technology, University of Chemistry and Technology Prague, Prague, Czechia; ^4^ Department of Inorganic Technology, Faculty of Chemical Technology, University of Chemistry and Technology Prague, Prague, Czechia

**Keywords:** bipyridine, bipyridinium, redox flow battery, property tuning, synthesis, electrochemistry

## Abstract

Bipyridinium salts are currently very popular due to their perspective applications in redox flow batteries. Hence, we designed and prepared a series of bipyridiniums based on 2,2′-, 3,3′-, and 4,4′-bipyridine and 2,2′-bipyrimidine. The straightforward synthesis utilizes commercially or readily available starting compounds and their direct *N*-alkylation, mostly using 1,3-propanesultone. All eleven target derivatives with systematically evolved structure were investigated by cyclic voltammetry, which allowed elucidating thorough structure-property relationships. The electrochemical behavior depends primarily on the parent scaffold, type of *N*-alkylation, number of quaternized nitrogen atoms, planarity, counter ion as well as the used media. Two derivatives featuring quasi-reversible redox processes were further tested on rotating disc electrode and in a flow battery half-cell. 4,4′-Bipyridinium derivative bearing two sultone residues showed better performance and stability in the flow half-cell with small capacity decays of 0.09/0.15% per reduction-oxidation cycle, based on the number of the utilized redox processes (one/two).

## Introduction

Bipyridines represent simple yet very interesting and tempting heteroaromatic scaffold with tremendous use across various research fields. In principle, we can distinguish six bipyridine derivatives that differ in the mutual orientation of both pyridine rings. The synthesis of parent bipyridines is well-known since 1950 (Summers, 1984). Some bipyridines occur naturally, for instance 2,2′-bipyridine is present in the petroleum or 2,3′-bipyridine can be found in the seedlings of tobacco (*Nicotiana species*) (Kallianos et al., 1972; Gray et al., 2008). Relatively small changes in mutual orientation of both bipyridine nitrogen atoms lead to significant structural differences. For instance, X-ray diffraction analysis revealed that both rings in 2,2′-bipyridine are coplanar with the nitrogen atoms in an *anti*-position. Accordingly, 4,4′-bipyridinium dication possesses planar arrangement (Summers, 1984). Bipyridine salts based mainly on viologen (*N*,*N*′-dialkyl-4,4′-bipyridinium) found manifold applications as active components in electrochromic devices (Monk et al., 1999; Alesanco et al., 2016), molecular machines (Coumans et al., 2013; Song et al., 2017), and redox-flow batteries (Beh et al., 2017; Hu et al., 2017). Recently, redox-flow batteries have gained significant attention due to their perspective to become low-cost energy storage device for large-scale applications. Especially methyl viologen (also known as herbicide paraquat) is used as popular anolyte in aqueous redox-flow batteries (Hu et al., 2017; Janoschka et al., 2016; Liu et al., 2016; Hu et al., 2018; Janoschka et al., 2015), which is mostly due to its well-defined electrochemical properties, relatively high solubility, and simple preparation. Divalent bipyridine cation may undergo a two-step reduction to the neutral molecule *via* a monovalent radical cation, which is often accompanied by a characteristic color change. Moreover, the redox potential and solubility of bipyridines can further be tuned by proper *N*-substitution and positioning of the nitrogen atoms. Viologen solubility issues can be solved by introduction of solubilizing groups such as 3-(trimethylammonium)propyl, 3-sulfonatopropyl, hydroxyalkyl or 3-phosphoniumpropyl, which also improves battery voltage and energy density (DeBruler et al., 2017; Jin et al., 2020; Liu et al., 2020). It is also believed that such structural tuning suppresses eventual dimerization of the reduced form (Hu et al., 2018). Viologen molecular assemblies with up to 15 viologen units were recently tested in RFB providing multi-electron transfer but also significant stability issues (Ohira et al., 2020).

**FIGURE 1:**
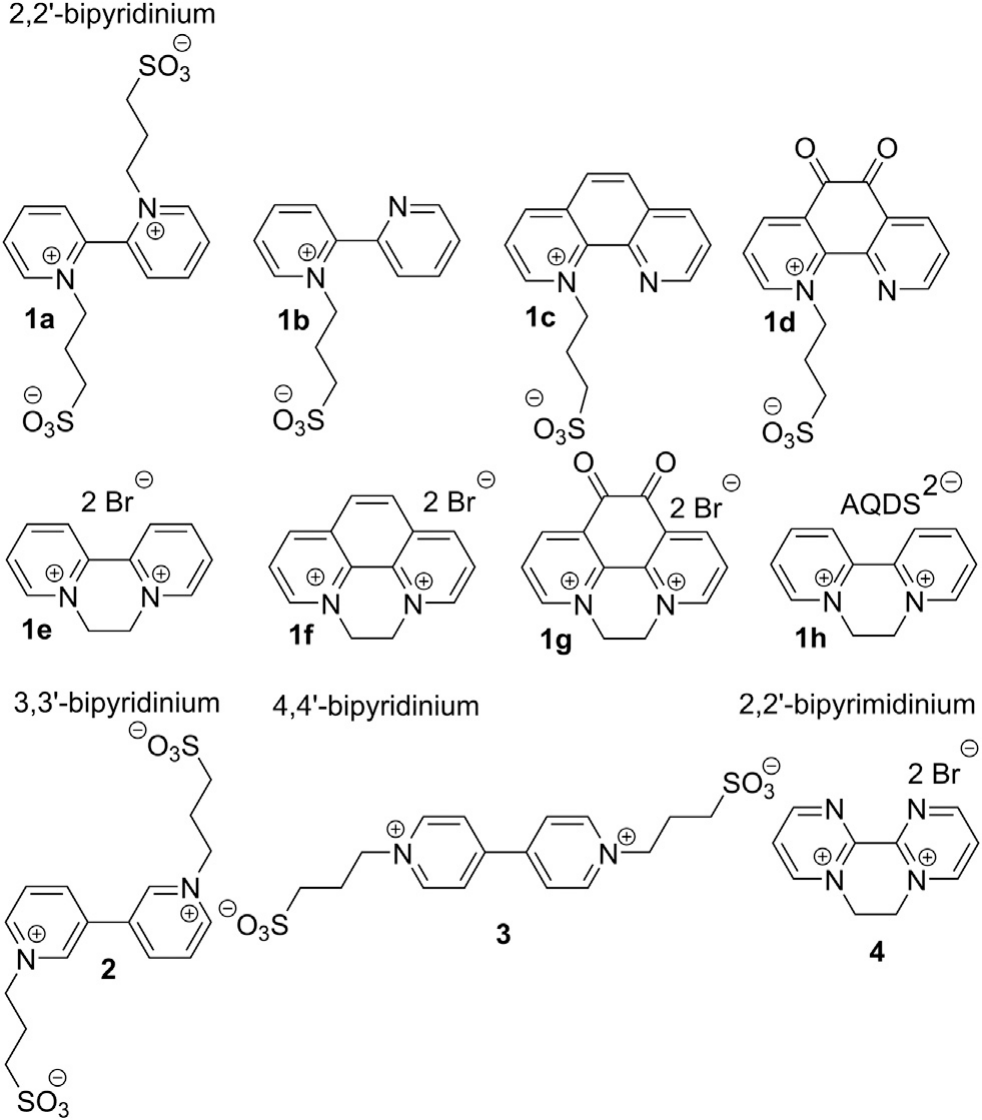
Molecular structure of the synthesized bipyri(mi)diniums.

Hence, we report herein on structural elaboration of bipyridine derivatives especially in view of tuning their electrochemical behavior. To the best of our knowledge, no such systematic study on bipyridinium salts has been carried out so far. We report herein preparation of eleven bipyri(mi)dinium salts 1–4 (Figure 1) and their further electrochemical studies. Three bipyridine isomers (2,2′, 3,3′, and 4,4′) were examined and further structurally tuned by various bridges (ethylene or oxalyl), *N*-substituents, and counter anions. Whereas 2,2′-bipyridines 1a–d are zwitterionic, 1e–h possess separate bromide or anthraquinonedisulfonate (AQDS) anions. 3,3′- and 4,4′-bipyridine derivatives 2 and 3 are positional isomers of 1a, while bipyrimidine 4 relates to 1e.

## Results and Discussion

### Synthesis

**SCHEME 1:**
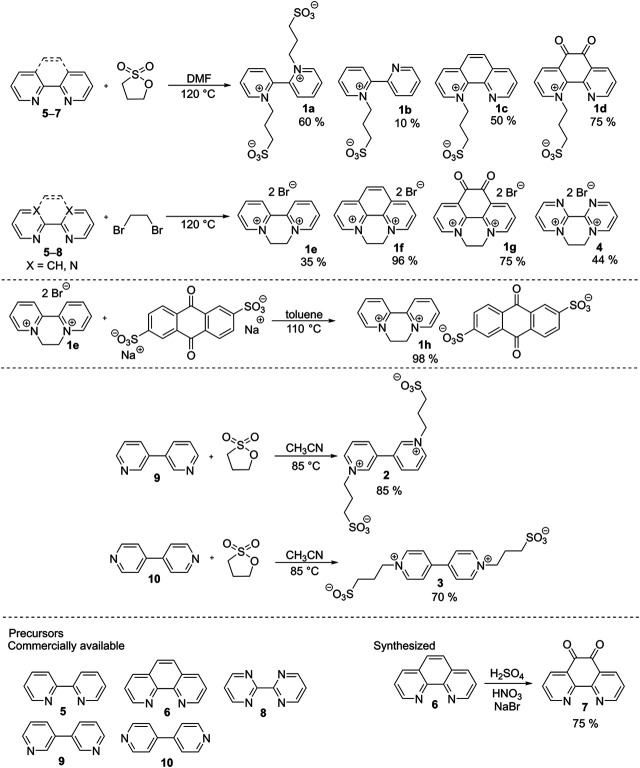
The synthesis of target derivatives 1a–h and 2–4.

The synthetic strategy toward bipyridines and their salts is outlined in Scheme 1. Starting materials 5, 6, 8, 9, and 10 are commercially available, while precursor 7 was prepared by oxidation of 1,10-phenantroline 6 using a mixture of sulfuric and nitric acids. One- or two-fold *N*-quaternization of 2,2′-bipyridines 5–7 with 1,3-propanesultone in *N*,*N*-dimethylformamide (DMF) at 120°C afforded 1a–d with the yields 10–75%. Depending on the amount of the sultone used, parent 2,2′-bipyridine 5 can be quaternized on one (1b) or both (1a) nitrogen atoms. On the contrary, phenantroline derivatives 6 and 7 underwent only mono *N*-alkylation, probably due to a steric hindrance. Repeated crystallization of phenanthroline derivative 1d proved to be tedious and inefficient, therefore, 1d can be prepared with only a limited purity (∼90%). The second series, consisting of ethylene bridged derivatives 1e–g and 4 with bromide counter ions, was synthesized from 5–8 and 1,2-dibromoethane (used as solvent). The attained yields range from 35 to 96%. The bromide anion in molecule 1e was further replaced by AQDS by its reaction with disodium 9,10-anthraquinone-2,6-disulfonate in toluene. Derivative 1h was isolated in almost quantitative yield. The synthesis of bipyrimidinium derivative 4 was similar and provided moderate 44% yield. 3,3′- and 4,4′-bipyridinium salts 2 and 3 were synthesized analogically to 1a by a two-fold quaternization using 1,3-propanesultone in acetonitrile at 85°C with good yields 85 and 70%, respectively. The synthesis, analytical data, and native spectra are given in the Supplementary Material.

**SCHEME 2:**
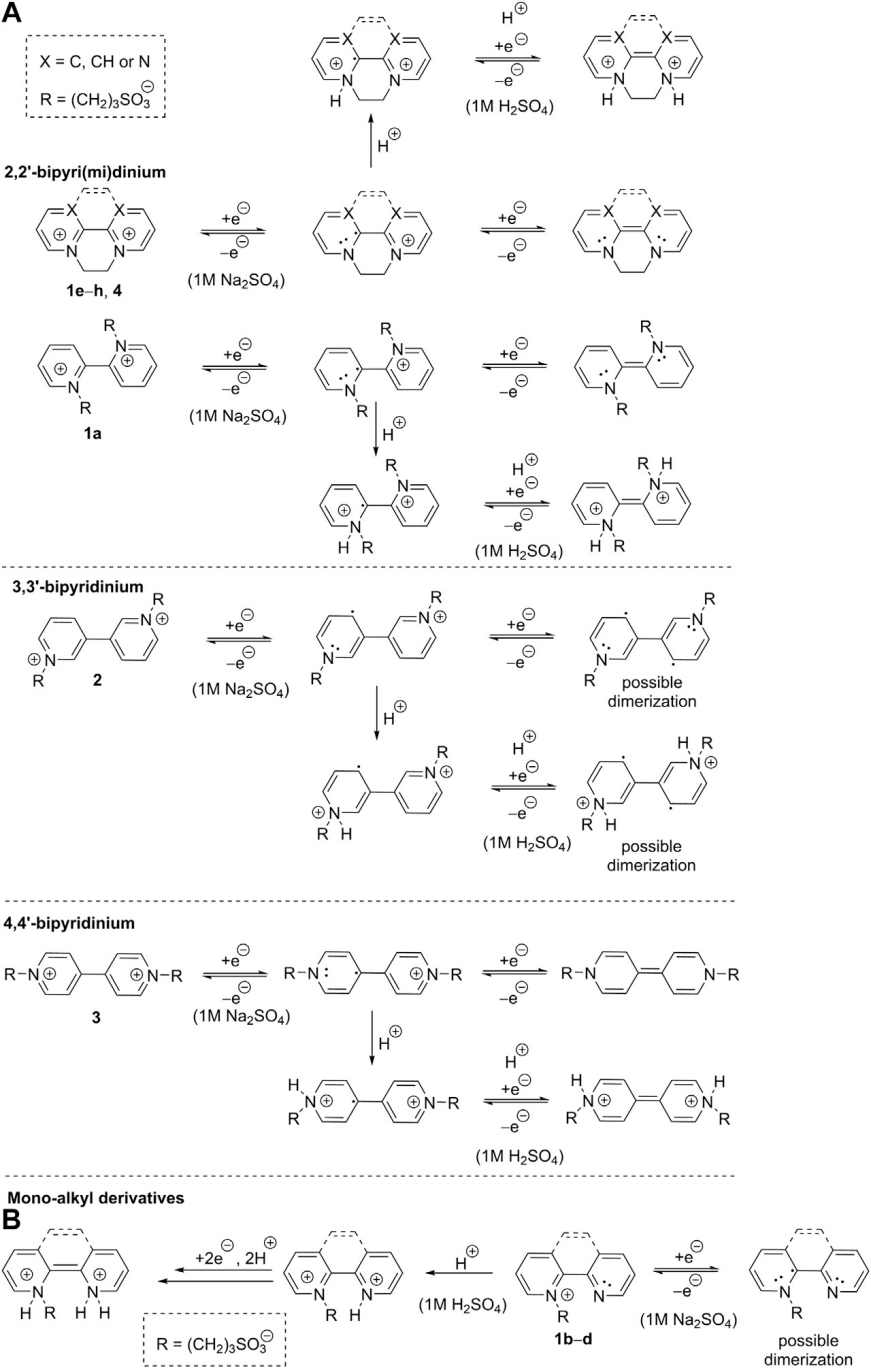
Proposed two-electron reductions of bipyri(mi)diniums 1a, 1e‐h, and 2‐4 **(A)** and one- and two-electron reductions of pyridiniums salts 1b‐d **(B)**.

### Electrochemical Characterization by Cyclic Voltammetry

All target compounds were intended as anolytes for aqueous RFB and as such, they possess pyridinium and carbonyl redox centers. Electrochemical redox processes involving these centers were preliminarily screened by cyclic voltammetry carried out in aqueous electrolytes – 1 M H_2_SO_4_ and 1 M Na_2_SO_4_ on activated glassy carbon electrode (Bystron et al., 2019). Investigated bipyri(mi)dium salts 1–4 proved to be unstable in alkaline media (1 M NaOH) as judged by their dark color and disappeared signals in ^1^H NMR spectra (see the Supplementary Material). The fundamental electrochemical data are gathered in Supplementary Tables S1 and S2; a full list of measured voltammograms is given in the Supplementary Figures S2–7. All potentials within this study are related to a standard hydrogen electrode (SHE).

**FIGURE 2:**
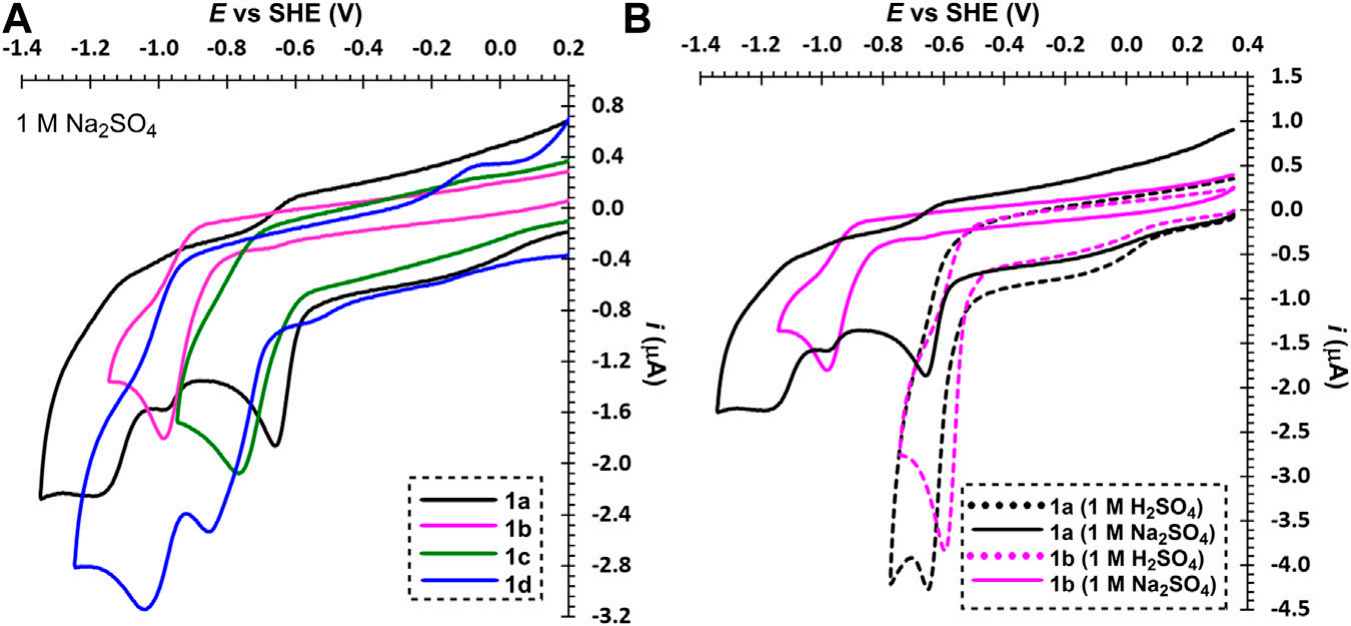
Electrochemical reductions of 2,2′-bipyridiniums 1a–d **(A)** and 1a and 1b in neutral and acid media **(B)**, *c* ∼ 1 mM.

In neutral electrolyte (Na_2_SO_4_), bipyridium 1a underwent two consecutive reductions (Figure 2A), which reflects presence of two pyridinium units and increased electron density upon the first reduction (formation of an amine). A reduction of the residual impurity (1b) can be partially observed at *E*_pc1_ = –0.97 V. Indeed, this mono-quaternized derivative showed one reduction with the potential lying between the two aforementioned redox processes seen for 1a. In both media are reductions of 1a–b irreversible, which is probably due to subsequent chemical processes (dimerization) or possible protonation in acid media (and re-oxidation *via* a different pathway). A similar behavior was observed for phenanthroline derivative 1c, which is even less soluble in both electrolytes. Oxalyl-bridged compound 1d showed two consecutive electrochemical processes in neutral media that belong to one-electron reduction of the pyridinium and the carbonyl group. Thus, the electron deficient pyridinium is reduced prior to the less polarized carbonyl function. Moreover, the re-oxidized species of 1c and 1d, generated at high overpotentials above 0 V vs. SHE, were strongly irreversible adsorbed at the electrode surface. This is probably due to a low solubility of 1c–d containing only one solubilizing group. It is obvious that electrochemically generated reduced forms of 1a–d are chemically instable in both media. An extension and planarization of the π-system as well as introduction of the carbonyl redox center shift the reduction potential to more positive values. This is however undesirable for anolyte and do not bring a desired improvement in chemical stability of the reduced form(s).

From the recorded voltammograms we can further deduce that mono-quaternized bipyridines 1b–d are in acid media (1 M H_2_SO_4_) clearly protonated on the pyridine nitrogen. This results in a bipyridinium with permanent and temporary quaternization (*N*-alkylation/protonation) as demonstrated by a comparison of 1a and 1b in acid and neutral media (Figure 2B). Whereas the first reduction potential of 1a is pH-independent, mono-quaternized 1b showed in acid media the first reduction potential shifted by 400 mV to more positive value (see also Supplementary Tables S1 and S2). Derivative 1b underwent one-electron reduction in neutral media and two-electron reduction in H_2_SO_4_. The second electron transfer is carried out at the potential corresponding to the first reduction; the respective peak corresponds to a two-electron reduction process. This is due to immediate protonation of the amine generated upon the first reduction step, which keeps the scaffold electronically identical to a state before the first reduction. A similar behavior has been observed for all mono-quaternized derivatives 1c–d. For the same reason, bipyridinium 1a underwent in 1 M H_2_SO_4_ a single two-electron reduction.

**FIGURE 3:**
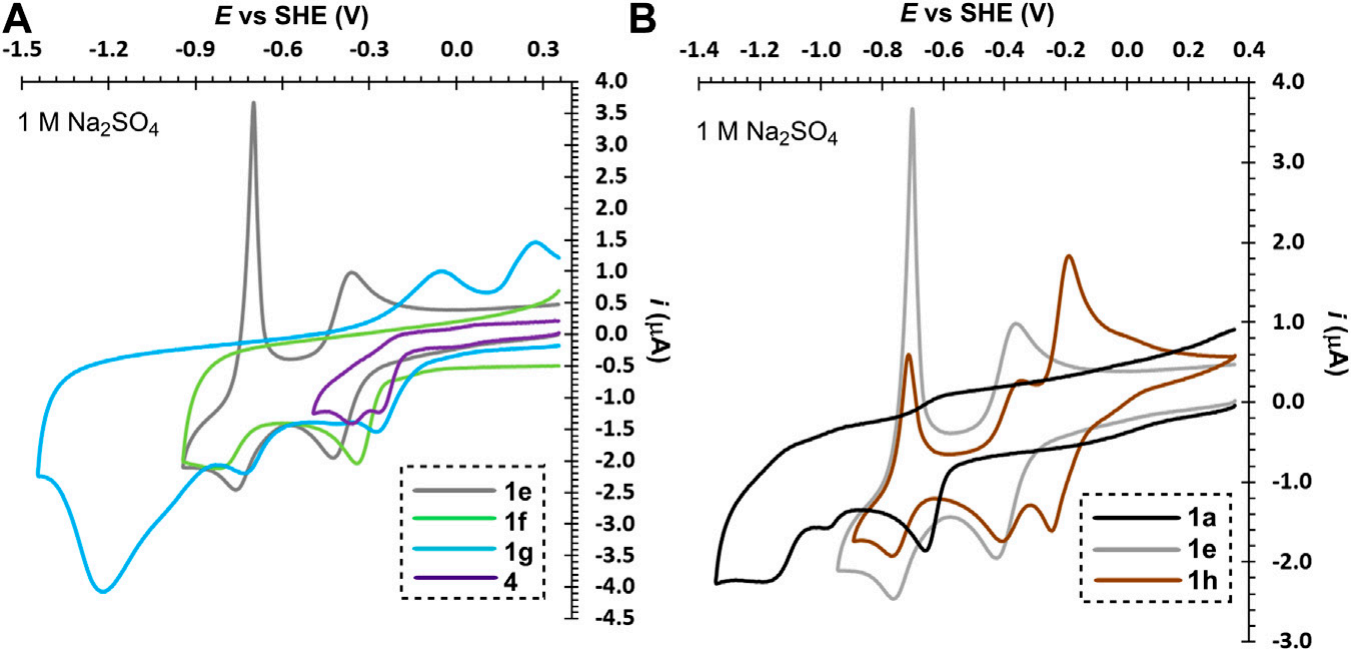
Electrochemical reductions of bridged bipyridiniums 1a–g and 4 **(A)** and influence of the counter ion and planarity in bipyridiniums 1a, 1e, and 1h **(B)**, *c* ∼ 1 mM.

The second series of molecules 1e–g (Figure 3A) possesses an additional ethylene bridge, which assures their permanent quaternization. The counter ion is Br^−^ with a quasi-reversible oxidation found at around +1.3 V. From the measured voltammograms is obvious that the reductions are almost independent on pH (Supplementary Tables S1 and S2). In both media, known diquat 1e underwent two consecutive one-electron reductions; similarly to 1a are both processes merged in 1 M H_2_SO_4_ (Figure 3A and Supplementary Figure S4A). The first reduction is always reversible regardless of the used media, whereas the second process in acid media is irreversible as the fully reduced basic form (an amine) undergoes protonation and cannot be re-oxidized over the same pathway. This protonation is suppressed in the neutral media, however, the fully reduced form is no more water-soluble and is being deposited on the electrode surface (a sharp peak of the re-oxidation). Despite is 1e capable to undergo two consecutive reversible reductions, its both redox centers are simultaneously solubilizing groups that are lost upon reduction. The water-insolubility of the fully reduced form limits its use as one-electron reducible RFB anolyte with lower volumetric energy capacity. Although interesting and reasonable, replacing bipyridinium scaffold with bipyrimidinium (1e→4) turned out to have very detrimental effect on the chemical stability of the reduced form (slightly more stable in acid media). Derivative 4 showed two following one-electron reductions and, as compared to 1e, is more water-soluble. The electrochemical behavior of compounds 1f and 1g is similar to those described for related derivatives 1c and 1d. Electrochemically reversible processes were in bipyridinium/bipyrimidinium series 1e–g/4 observed solely for diquat 1e and its further structural tuning was rather counterproductive. Considering the whole series 1a–g as well as compound 4, the performed structural variation affected mostly chemical stability and solubility of the generated reduced forms.

Subsequently, we focused our attention toward the counter ion effect. The bromide ion in diquat 1e was replaced by AQDS, well-known anolyte in RFB, to gain hybrid derivative 1h. The voltammograms in Figure 3B clearly show that beside the bipyridinium reduction, we observed also reversible reduction of the quinone system of AQDS. Whereas the position of the pyridinium reduction peaks is almost independent on pH (Supplementary Figure S1), the AQDS reduction is affected significantly as the AQDS redox processes involve either two or one proton(s) in acid/neutral media (Huskinson at al., 2014). A further comparison of rigid 1e/1h and nonplanar 1a reveals irreversible reduction of the latter. Hence, the planarity of the main π-conjugated scaffold as well as *anti*-arrangement of both nitrogen atoms seem to be significant parameters affecting electrochemical reversibility of 2,2′-bipyridines.

**FIGURE 4:**
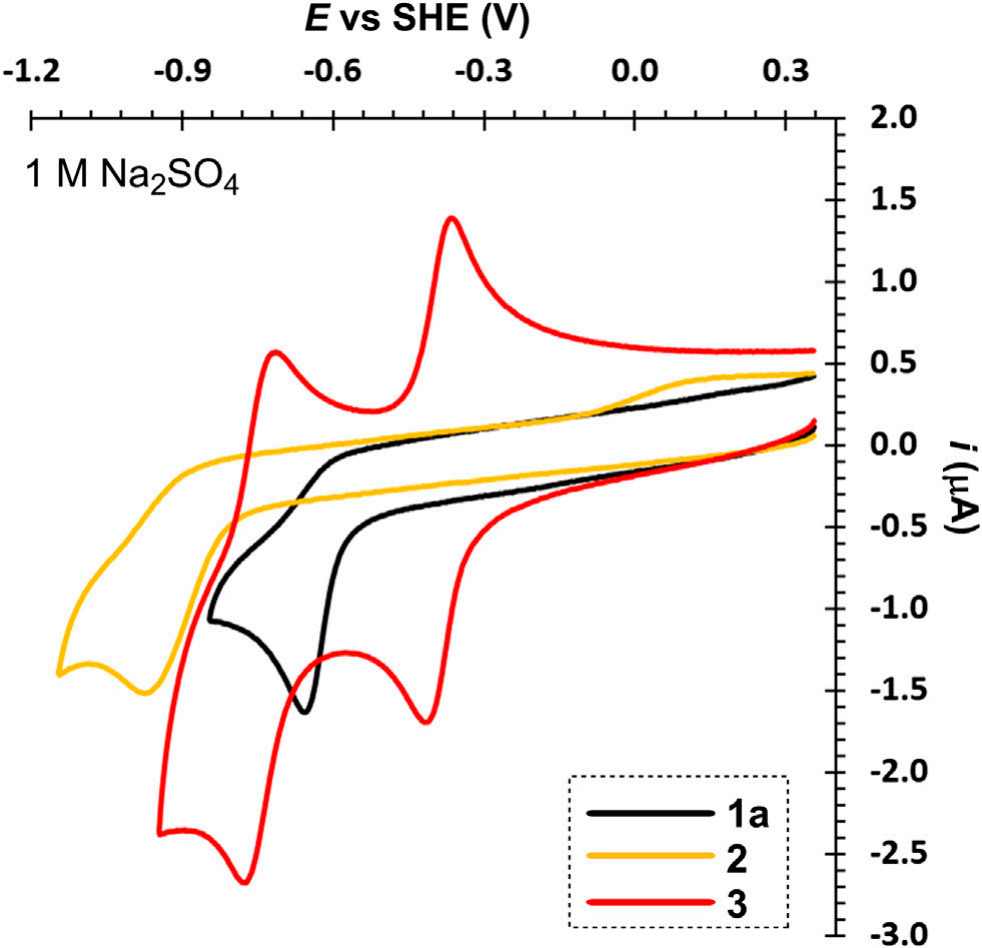
A comparison of 2,2'- (1a), 3,3'- (2), and 4,4′-bipyridinium’s (3) voltammograms, *c* ∼ 1 mM.

Figure 4 compares three particular bipyridinium salts that differ in mutual orientation of the rings (nitrogen atoms). Derivative 3, related to paraquat, showed two, reversible, one-electron reductions, which is in line with the published data (Liu et al., 2018). Its planar structure with remote alkyl chains preventing their repulsion allows efficient conjugation of both pyridinium rings. In contrast to 1e and due to two sulton residues, its fully reduced form is still soluble in water. Hence, in neutral media, both reduction steps can be utilized without a precipitation and deposition at the electrode surface. On the other hand, protonation of the fully reduced form in acid media prevents utilization of the second reduction step. When going from 4,4′- to 2,2′- and 3,3′-bypiridinium (3→1a→2), the first reduction potential shifts to more negative values. More importantly, this shift is accompanied by a loss of reversibility. Both nitrogen atoms in 2 are localized out of alternating positions and, despite the lowest potential of the first reduction, the reduction product is chemically unstable and may undergo various consequent processes. On the contrary, both nitrogen atoms in 1a are alternating but proximity of two C = N^+^ polar bonds makes 1a prone to attack of hydroxide anion at basic conditions thus lowering its stability.

From the aforementioned electrochemical observations, we can propose a mechanism of two-electron reduction of bipyri(mi)diniums 1a, 1e–h, and 2–4 (Scheme 2A). In general, the first reduction of all derivatives generates radical cations with possible resonant stabilization, which partially suppress their dimerization. For 2,2′- and 4,4′-bipyridiniums, the second reduction affords quinoid arrangement, while this is not the case of 3,3′-bipyridinium. The latter possesses both nitrogen atoms in non-alternating positions and, therefore, its two-electron reduction produces diradical with diminished chemical stability. The reducible centers of 1e–h and 4 are simultaneously solubilizing groups that are lost upon reduction. Hence, their two-electron reduction produces uncharged intermediates that are sparingly soluble in aqueous neutral media. On the contrary, derivatives 1a, 2, and 3 with sultone pendants are well soluble in water regardless their redox state. The (electro)chemical stability, the number of generated reduced forms, and the position of the redox potential are further influenced by the planarity, presence of the ethylene/oxalyl bridge, the number of *N*-substituents, and the used media. Dialkyl derivatives 1a, 1e–h, and 2–4 are reduced in different way in acid media. Both reductions produce tertiary amines that are immediately protonated to afford ammonium salts (Scheme 2A). A mechanism of reduction of monoalkyl derivatives 1b–d is outlined in Scheme 2B. In neutral media, one-electron reduction takes place accompanied by a formation of unstable radical that may undergo further chemical processes. Pyridine ring of 1b–d is quickly protonated in acid media to afford dication and the subsequent two-electron reduction correspond to the aforementioned mechanism shown in Scheme 2A.

### Electrochemical Characterization on Rotating Disc Electrode (RDE)

The selected compounds 1e and 3 exhibiting chemically reversible redox behavior in neutral environment were subsequently characterized on glassy carbon RDE in 1 M Na_2_SO_4_; see the Supplementary Material for detailed description of the used methodology. The measured curves together with their evaluation are presented in Supplementary Figures S8–11. Levich and Koutecký-Levich analysis of the measurements revealed kinetic parameters and diffusion coefficients that are summarized in Supplementary Table S3. In accordance with the results of preliminary CV measurements described in the previous section both compounds exhibited two subsequent one electron quasi-reversible redox processes in a broad range of potential scan rates (5,000–10 mV s^−1^), see Supplementary Figure S8. The heterogeneous rate constant *k*° was evaluated from RDE measurement at various rotations (500–3,000 rpm) using Koutecky-Levich method. For the first redox step it was 7.9 and 15.1 × 10^–3^ cm s^−1^ for 1e and 3, respectively. In case of 3 the obtained value is significantly lower than the reported values for the identical compound in neutral electrolyte (0.28 cm s^−1^) (DeBruler et al., 2018). The discrepancy between the values is most probably caused by inaccuracy of Nicholson method, which was used for the estimation of *k*° in the cited study. Nevertheless, the value of *k*° are still by orders of magnitude higher than in case of mostly exploited vanadium redox couples (1.7 × 10^–5^ cm s^−1^ for V^3+^/V^2+^ in acidic electrolyte) (Sum and Skyllas-Kazacos, 1985). The fast electrode reaction kinetics of the bipyridine-based molecules is promising for their application in redox flow battery, in particular, with respect to low activation polarization of the battery. The diffusion coefficient evaluated from the same RDE measurements were 2.9 and 2.5 × 10^–6^ cm^2^ s^−1^ for 1e and 3, respectively. These results indicate that the difference in structure of both molecules does not cause big differences in their diffusivities. For compound 3 the achieved value is fully in line with the previously reported values (DeBruler et al., 2018). The high values of *D* are required for the efficient battery operation at high current densities, where the efficiency losses of the battery related to mass transport of active species (reactants) to the electrode surface are dominating.

### Electrochemical Stability in Flow Battery Half-Cell

**FIGURE 5:**
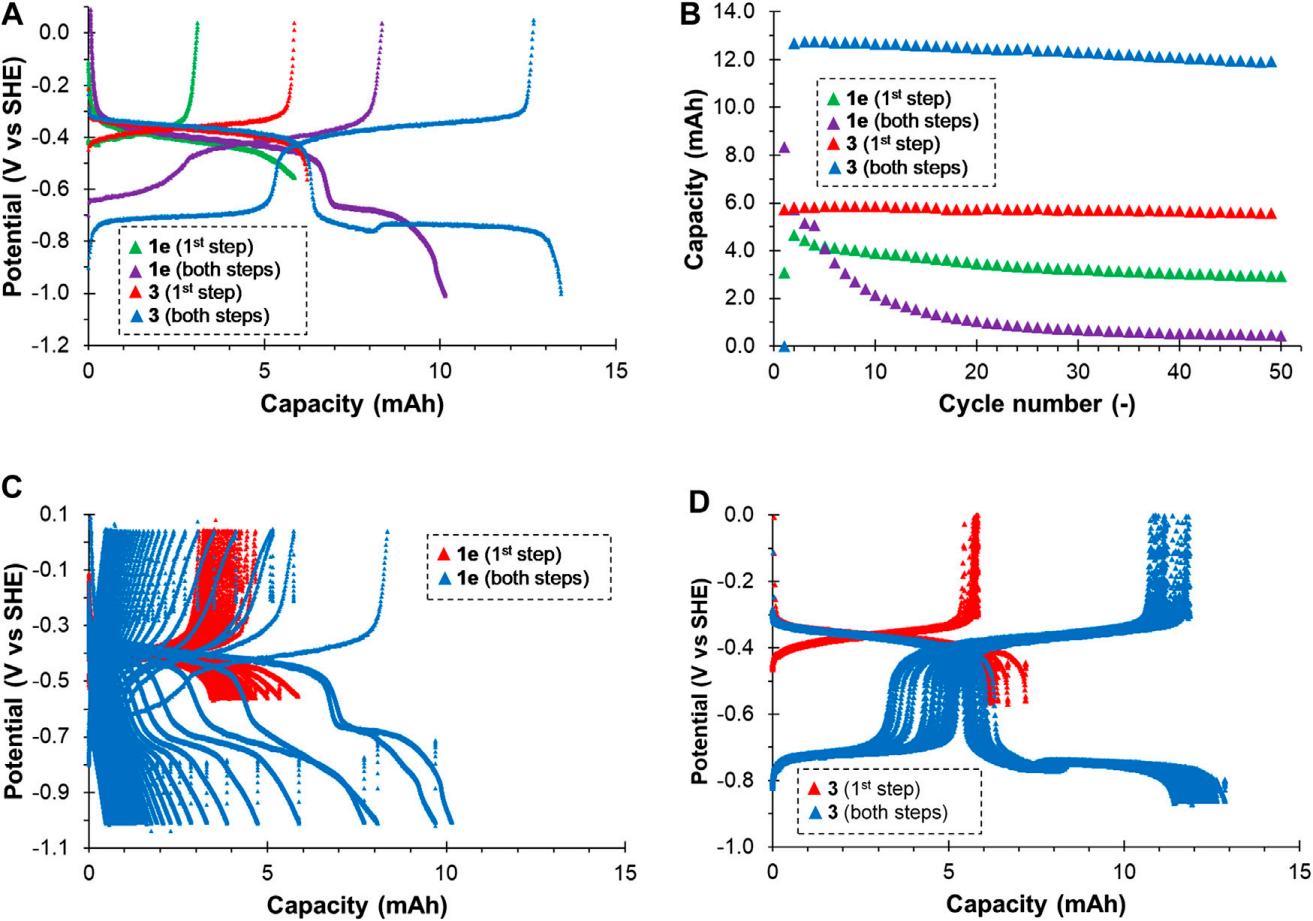
Galvanostatic reduction-oxidation cycling of neutral electrolyte based on bipyridiniums 1e and 3 at 12.5 mA cm^−2^, 20°C: Development of working electrode potential of electrolysis cell during the first cycle **(A)**; Evolution of oxidation (discharge) capacity during entire cycling **(B)**; Development of working electrode potential of electrolysis cell during entire cycling for 1e **(C)** and 3 **(D)**.

The compounds 1e and 3 exhibiting quasi-reversible electrochemical behavior on glassy carbon were subsequently tested in a flow battery cell in 3-electrode set-up using thermally treated graphite felt working electrode. The 25 mM solution of the active compound in neutral supporting electrolyte was galvanostatically cycled in a potential window corresponding to the first and both redox steps, while hydrogen and oxygen evolution from the supporting electrolyte proceeded on a counter electrode separated from the working electrode by an ion-exchange membrane preventing cross-over of active compound through the membrane. We used anion-exchange membrane for cationic 1e, while for zwitterionic 3, high equivalent weight cation-exchange membrane was employed. Negligible permeation of both active compounds was confirmed by UV-Vis analysis of counter electrolyte after the cycling. Moderate current density of 12.5 mA cm^−2^ and relatively high electrolyte flow rate of 40 ml min^−1^ were used to minimize mass transport polarization of the working electrode. The parameters evaluated from the battery cycling of both compounds are summarized in Table 1. The reduction-oxidation potential profile is shown in Figure 5. For both compounds we can see two reduction and oxidation plateaus corresponding to the first (at approx. −0.4 V vs. SHE) and second step (at approx. −0.7 V vs. SHE), which agrees well with the aforementioned CV measurements on glassy carbon. However, both compounds behaved differently in terms of capacity utilization (*CU*), Coulombic efficiency (*CE*), and stability upon cycling. In case of 1e we observed relatively low values of *CU* and *CE* and fast capacity decay (2.5% *Q*_theo_/cycle leading to almost 60% capacity loss within 50 cycles) when cycled within both redox steps. This decay was most probably caused by deposition of the fully reduced (and thus non-ionic) 1e on the surface of graphite felt electrode, which has formerly been reported for similar derivatives (Bird and Kuhn, 1981). Smaller capacity decay was observed for cycling in the first redox step region (0.7% *Q*_theo._/cycle leading to 26% capacity loss within 50 cycles). As reported for bipyridine-based compounds (Bird and Kuhn, 1981), this can be attributed to dimerization, chemical reaction or disproportionation of partially reduced bipyridine radical leading to deposition of insoluble fully reduced form.

**TABLE 1:** Parameters evaluated from the galvanostatic cycling of electrolytes in flow electrolysis cell.

Comp./Param.	*Q*_theor._ [mAh]	*Q*_dis_ ^a^ [mAh]	*CU* [%]	*CE* ^b^ [%]	d*Q*_rel_ ^c^ [%]	Δ*Q*_rel._ ^d^ [%]
1e (1st step)	6.7	4.7	69	94	–0.70	25.7
1e (both steps)	13.4	8.4	62	86	–2.50	58.9
3 (1st step)	6.7	5.9	87.5	94.2	–0.09	4.2
3 (both steps)	13.4	12.8	95.3	94.3	–0.15	6.2

^a^ Maximal discharge (oxidation) capacity obtained during cycling.

^b^ Averaged value from the 10th–20th cycle.

^c^ Evaluated from slope of capacity-cycle number dependence from the 10th–50th cycle.

^d^ Evaluated from difference between capacity of the 1st and 50th cycle.

On the contrary, compound 3 showed significantly better performance and stability in both potential ranges. Very small capacity decay of 0.09% *Q*_theo._/cycle leading to 4.2% overall capacity decay after 50 cycles was observed for the first reduction step. Slightly higher decay was observed when cycled in both reduction steps: 0.15% *Q*_theo._/cycle leading to over 6% loss of the theoretical capacity within 50 cycles.

### NMR *Post Mortem* Analysis

The redox processes of 1e and 3 were also monitored by ^1^H nuclear magnetic resonance (NMR), see the Supplementary Material for detailed description. The NMR *post mortem* analysis of bipyridinium 1e after cycling in the battery half-cell showed significant decrease of the signals corresponding to initial compound when cycled within the first step (around 70% loss of material, Supplementary Figure S12, Spectrum 2) and complete loss of the initial compound when cycled in both steps (Supplementary Figure S12, Spectrum 3). Detailed inspection of the ^1^H NMR Spectrum 3 revealed the presence of ca. 20% of broad signals, hinting formation of insoluble polymeric materials. Attempted extraction of the organic material from the electrode with DMSO gave no results. Hence, polymeric water-insoluble compounds were probably formed under the experimental conditions. Analogous *post mortem* ^1^H NMR spectroscopy analysis of 3 showed around 18% loss of the starting compound 3 after 50 cycles at the first step (Supplementary Figure S13, Spectrum 2) and surprising complete disappearance of signals after cycling in both steps (Supplementary Figure S13, Spectrum 4). This is in contrast with observed electrochemical data showing negligible capacity fade of the battery and can be explained by analyzing pH of the mixtures. We found that both samples were quite basic (pH ∼ 12), which is due to formation of an amine upon both reduction steps (see Scheme 2). A solution of structurally related piperidine in aqueous Na_2_SO_4_ of similar concentration possesses pH 12.3. Under such alkaline condition, we assumed that reversible reaction of 3 with hydroxide anion is responsible for blurring the NMR signals (see Supplementary Material for extended discussion). Indeed, mild acidification of the mixtures with NaH_2_PO_4_ led to reappearing of the NMR signals. When the electrochemical experiments were performed in the presence 0.1 M Na_2_HPO_4_/NaH_2_PO_4_ buffer, no blurring of the signals was observed. However, we observed about 20% loss of the starting compound 3, probably due to decomposition under basic condition (see Supplementary Figure S13, Spectra 3 and 5).

## Conclusion

A series of bipyri(mi)dinium derivatives with systematically evaluated structure has been designed and synthesized. These represent a popular class of salts applicable in aqueous redox flow batteries. Based on the aforementioned discussion, the following outcomes and structure-property relationships can be summarized/elucidated:

- The straightforward synthesis involves *N*-alkylation; the reaction with 1,3-propanesultone seems to be the most useful strategy.
- The fundamental electrochemical properties of bipyri(mi)dinium salts is affected by the number of quaternized nitrogens (one- or two-step(s) reduction). Due to anticipated higher capacity, two pyridinium centers are preferred for application in RFB.
- Bipyridinium regioisomers with different mutual orientation of the nitrogen atoms (2,2′, 3,3′, and 4,4′) affect mostly the reversibility of the redox processes (repulsion of *N*-alkyl groups and non-conjugate arrangement). 4,4′-Bipyridine is preferred as a parent scaffold.
- 3-Sulfonatopropyl proved to be excellent solubilizing group as it brings permanent water solubility regardless the redox state of the used bipyridinium. Two *N*-alkyls are generally preferred.
- Further bridges such as oxalyl, ethylene or ethenylene planarizes the p-system but shift the reduction potential to more positive values and do not improve stability of the reduced form.
- The counter ion must also be considered, as bromide is electrochemically active but may be easily replaced with AQDS, which brings additional redox centers. Zwitterionic arrangement, as present in sultone derivatives, is preferred.
- The used media is another important aspect. Whereas bipyri(mi)dinium salts proved to be unstable in alkaline media, neutral or acid media significantly affect reversibility of the redox process (possible protonation of the intermediate amines).
- Detailed electrochemical characterization of compounds 1e and 3 on glassy carbon rotation disc electrode revealed fast reaction kinetics of both compounds and comparable values of diffusion coefficient.
- Investigation of compounds 1e and 3 in flow battery half-cell revealed much better electrochemical stability and Coulombic efficiency upon galvanostatic cycling of the latter. It features a trade-off between all desired structural aspects listed above.
- The performed NMR *post mortem* analysis corroborates the electrochemical outcomes and revealed significant electrolyte alkalization accompanied by a loss of material. This is due to formation of an amine upon the first and the second reduction. This obstacle may be partially suppressed by buffering the solution.

## Data Availability

The original contributions presented in the study are included in the article/Supplementary Material, further inquiries can be directed to the corresponding authors.
